# Supplementary material for: Hypermethylation of DNA Methylation Markers in Non-Cirrhotic Hepatocellular Carcinoma
Source: Cancers (Basel). 2023 Sep 28;15(19):4784. doi: 10.3390/cancers15194784 (PMC10571582; doi:10.3390/cancers15194784)
Supplement: Supplementary file 1 [file cancers-15-04784-s001.zip › cancers-2580713-supplementary.pdf]

Table S1. Patients' information for DNA methylation analysis using qMSP (tissues)

	Non-cirrhotic HCC	Hepatitis	Benign lesions	Cirrhotic HCC	Cirrhosis	Total
**Sex**						
Male	33	23	1	10	22	89
Female	27	11	6	5	8	57
**Age**						
<40	4	10	1	1	5	21
[40-59]	22	13	6	5	16	62
≥60	34	11	0	9	9	63
**Etiology**						
HBV (viral)	7	4	0	6	6	23
HCV (viral)	1	3	0	2	3	9
NAFLD (non-viral)	16	4	0	1	0	21
ALD (non-viral)	8	11	0	4	15	38
Cryptogenic	28	12	0	2	6	48
Adenoma/FNH	0	0	7	0	0	7
Total	60	34	7	15	30	146

Abbreviations: qMSP, quantitative methylation-specific PCR; HCC, hepatocellular carcinoma.

Table S2. DNA methylation-specific primer and probe sequences

Gene	Detection	Oligonucleotide	Sequence 5' to 3'	Amplicon length (bp)	Annealing temperature (°C)
HOXA1	Exon 1 methylation	Forward	TTTACGTTTGTTTTTTGATTTAACGC	131	60
		Reverse	CCGACTACCTACCAAACTTCCG		
		Probe	CGTATTGAAGTTTTGTGAGTTA (MGB*)		
CLEC11A	Exon 3 methylation	Forward	TTAGGGGTTGCGGTAGTTGC	71	60
		Reverse	ACGCCTCCTACAAAACTTACACG		
		Probe	CGCGGTAGGCGATAT (MGB*)		
AK055957	Exon 1 methylation	Forward	TTCGGGTCGTTTTCGTTTAGAC	82	60
		Reverse	CTCCTCCGACGACAACCG		
		Probe	CGTATTGAAGTGAGTTCG (MGB*)		
TSPYL5	Exon 1 methylation	Forward	TTTAGGGGGAGTCGGTAGGC	81	60
		Reverse	GAAATAAAATAACCTAAAAACCGCTACG		
		Probe	TTTTTTTTTCGAGTCGGAGGAGTTGCG		
ACTB	Bisulfite conversion	Forward	AACCAATAAAACCTACTCCTCCCTTAA	133	60
		Reverse	TGGTGATGGAGGAGGTTTAGTAAGT		
		Probe	ACCACCACCCAACACACAATAACAAACACA		

*MGB minor groove binder, bp base pairs. Abbreviations: qMSP, quantitative methylation-specific PCR; HCC, hepatocellular carcinoma; HOXA1, homeobox A; CLEC11A, C-type lectin domain containing 11A; TSPYL5, Testis-Specific Y-encoded-Like Protein 5; ACTB, beta (β)-actin.

Table S3. MeD-seq on ALD-related liver disease and benign lesions (tissues)

	Non-cirrhotic HCC	Hepatitis	Benign lesions	Cirrhotic HCC	Cirrhosis	Total
**Sex**						
Male	5	7	1	3	5	21
Female	3	3	6	1	10	23
**Age**						
<60	3	6	7	0	10	26
≥60	5	4	0	4	5	18
**Tumor size***						
≤5 cm	1	NA	NA	2	NA	3
>5 cm	6	NA	NA	2	NA	8
**Etiology**						
ALD	8	10	0	4	15	37
Adenoma/FNH	0	0	7	0	0	7

* 1 non-cirrhotic HCC patients without information about tumor size. Abbreviations: MeD-seq, Methylated DNA sequencing; HCC, hepatocellular carcinoma; FNH, focal nodular hyperplasia; ALD, alcoholic liver disease; NA, not available.
